# Impact of Daily Grazing Time on Dairy Cow Welfare—Results of the Welfare Quality^®^ Protocol

**DOI:** 10.3390/ani8010001

**Published:** 2017-12-22

**Authors:** Kathrin Wagner, Jan Brinkmann, Solveig March, Peter Hinterstoißer, Sylvia Warnecke, Maximilian Schüler, Hans Marten Paulsen

**Affiliations:** Thünen Institute of Organic Farming, Trenthorst, 23847 Westerau, Germany; jan.brinkmann@thuenen.de (J.B.); solveig.march@thuenen.de (S.M.); peter.hinterstoisser@thuenen.de (P.H.); sylvia.warnecke@thuenen.de (S.W.); maximilian.schueler@thuenen.de (M.S.); hans.paulsen@thuenen.de (H.M.P.)

**Keywords:** dairy cows, animal welfare, grazing, pasture, Welfare Quality^®^

## Abstract

**Simple Summary:**

It is often presumed that grazing dairy cows experience better welfare than those that are housed all year round. But is this really the case? In this study, we wanted to find out whether the daily amount of time cows spent on pasture affected their welfare. We used the Welfare Quality^®^ assessment protocol for dairy cattle to measure cow welfare on 32 farms (organic and conventional) once in winter (=housing period) and once in summer (=grazing period, if provided). Farms were grouped according to daily grazing time (‘minor/zero’, ‘medium’, and ‘high’). In farms with grazing, overall welfare improved from winter to summer, whereas the situation in minor/zero grazing farms remained largely unchanged. While we found no overall effect of the amount of daily grazing time on cow welfare, the individual measures “% of cows with hairless patches” and “% of lame cows” received better scores in the high grazing farms. However, other measures e.g., related to water provision, scored worse in the grazing farms in summer as opposed to winter. We conclude that grazing offers a high potential to enhance dairy cow welfare during summer. However, beneficial effects are not guaranteed when the overall management does not satisfy the cows´ needs.

**Abstract:**

Grazing provides livestock better opportunities to act out their species-specific behavior compared to restrictive stable conditions. The aim of the present study was to examine the effects of daily grazing time on welfare of dairy cows in organic and conventional farms based on the Welfare Quality^®^ assessment protocol for dairy cattle (WQ^®^). Therefore, we applied the WQ^®^ on 32 dairy farms (classified in 3 groups: Group 0, minor/zero grazing, n = 14; Group 1, medium grazing, n = 10; Group 2, high grazing, n = 8). We assessed the status of animal welfare once in winter and once in summer. For statistical analyses we used mixed models for repeated measures, with group, season, and their interaction as fixed factors. At the WQ^®^ criteria level, five out of nine examined criteria improved in farms with grazing between winter and summer. In contrast, the welfare situation in minor/zero grazing farms remained largely unchanged. At the level of WQ^®^ measures, only the individual parameters “% of cows with hairless patches” and “% of lame cows” were affected positively by high grazing. Grazing offers a potential to enhance welfare of dairy cows during the summer season, while beneficial effects are not guaranteed when management does not satisfy the animals´ needs.

## 1. Introduction

In general, housing with access to pasture is viewed by the public as an animal-friendly and desirable form of keeping cows [1]. It is known that there are positive effects of providing pasture on welfare for dairy cows (for review [2]). For example, access to pasture provides better opportunities for livestock to act out their species-specific behavior compared to the restrictive stable conditions [3]. Cows at pasture showed more herd synchronization [4], spent more time lying [5] and showed less agonistic behavior [6] in contrast to confined animals. These behavior patterns are part of one of the five freedoms [7] to ensure animal welfare, namely “freedom of expressing normal behavior.” Furthermore, previous studies on various health parameters indicated for instance less lameness in systems with grazing [5,8,9], a reduced number of integument lesions [10,11], and decreased incidence of mastitis [12,13] compared to stable housing. These health parameters are in line with “freedom of pain, injury and diseases” [7] and provide evidence for improved welfare in systems with access to pasture.

However, previous studies mostly focused on particular impacts of grazing on behavior, health or certain diseases. Studies aiming to analyze the effect of grazing on the overall animal welfare status in cattle are rare. Olmos et al. [14] investigated several indicators in Ireland and found a tendency of better animal welfare for cows with access to pasture. Burow et al. [15] measured 17 different animal-related and resources-related indicators based on the Welfare Quality^®^ assessment protocol for cattle [16] (WQ^®^) in Denmark and determined a better welfare situation for dairy cattle in the summer grazing period compared to the continuous winter indoor housing. Additionally, former studies did not consider different daily grazing times (i.e., daily amount of time cows spent on pasture) when assessing welfare overall. An exception is one study from Germany covering one federal state (Lower-Saxony, 61 dairy farms; [17]). The authors concluded that grazing may have positive effects on animal welfare since farms with higher daily grazing times (6–10 h and >10 h per day) had better scores with respect to the WQ^®^ principles “good housing“ (mainly constituted by indicators of lying comfort) and “good health“ (due to absence of injuries) compared to farms with lower daily grazing times or zero grazing. Therefore, we aimed to examine the effects of different daily grazing times on welfare of dairy cows throughout Germany, on both organic and conventional farms. We hypothesized that overall cow welfare would improve from winter to summer on farms with grazing and that this improvement would be more evident with higher daily grazing times and that these effects can be found by applying the WQ^®^ assessment protocol for dairy cattle. We expected positive effects of grazing in the WQ^®^ criteria “comfort around resting,” “absence of injuries,” “absence of disease,” “expression of social behavior,” and “positive emotional state.”

## 2. Materials and Methods

This work was done within the project entitled “Increasing Resource Efficiency by Optimizing Crop and Milk Production on Whole Farm Level under Consideration of Animal Welfare Quality Aspects” (www.pilotbetriebe.de). Therein, 38 dairy farms in various climate zones and soil zones in Germany (Bavarian Tertiary Hill Country and Allgaeu, the North Sea and Baltic Sea coastal areas, the Rhine basin, the Westphalian basin and low mountain areas, and the East German inland area) are analyzed based on whole farm approaches. Besides regional aspects, selection criteria for entering the farm network were full-time farming, adjacent organic/conventional partner farm, data availability, and cooperation, as well as covering the bandwidth of productivity [18,19]. The multi-criteria selection approach that was required to reach the diverse goals of the project resulted in unbalanced data sets for several parameters. Altogether in Germany, there are 69,170 farm holdings with 4.2 million dairy cows. About 1.75 million cows have access to grazing areas (Destatis 2010). For the current study, we could assess the animal welfare on 32 (15 organic; 17 conventional) dairy farms twice per farm, using the Welfare Quality^®^ assessment protocol for dairy cattle [16]. Additionally, data from the milk recording scheme (MLP) and the German central data base on identification and information on animals (HI-Tier) were recorded. In addition, interviews were conducted and housing conditions were recorded. The mean herd size was 114 (30–726) dairy cows at the time of the winter survey and 123 (24–661) in summer. In 2014/2015, the average annual milk yield per cow was 6448 (3456–8079) kg in organic farms and 8273 (5437–9653) kg in conventional farms, respectively. In all herds, with the exception of one organic farm using hay, grass silage and/or maize silage were provided all year long and in addition to grazing. In the conventional herds, up to half of the mean yearly ration consisted of maize silage (11–52%), in the organic ones this share was 0 to 25%. Furthermore, concentrates were fed in all herds depending on daily milk yield. To assess the effects of the daily grazing time on the WQ^®^ measures and WQ^®^ criteria, the farms were grouped according to their daily grazing time (see Table 1).

### 2.1. Assessment of Animal Welfare

On the basis of the five freedoms defined by the Farm Animal Welfare Council 1992, animal welfare criteria and their assessment were defined by the European research project Welfare Quality^®^. WQ^®^ uses a “bottom-up” approach and was developed for a comprehensive on-farm assessment of animal welfare. In the first step, approximately 30 animal-related measured variables are used, which are then combined into twelve criteria. These criteria are then aggregated into four principles. In the fourth step, an overall welfare score is awarded. According to WQ^®^, a value of 100 corresponds to the best and a value of 0 to the worst of all possible values [20].

On each farm, the full WQ^®^ assessment protocol for dairy cattle [16] was performed twice: once during winter and once during the following summer.

Parameters concerning diseases were generated from MLP, HI-Tier, interviews, and animal-related measures. Thus, cows with somatic cell count above or equal 400,000 were counted for “% of cows with mastitis” from the MLP (as defined by WQ^®^). “Percentage of mortality” was calculated from HI-Tier. The parameters “% of dystocia” and “% of downer cows” as well as information regarding management (e.g., dehorning) were collected during the interviews.

In the winter season, all other WQ^®^ data were recorded in the barn, i.e., resource-based measures (e.g., length of water troughs and number of water bowls), assessment of animal condition (cleanliness, body condition, integument alteration, and lameness), avoidance distance, qualitative behavior assessment (QBA; defined by 20 terms of body language), social behavior (butts and displacements), and lying behavior (duration of lying down, lying outside the lying area, and lying down with collision). In the summer season, water supply, lying behavior, and QBA (criterion “positive emotional state”) were recorded on pasture (Groups 1 and 2). In addition, social behavior was observed on pastures for Group 2 in summer. All other WQ^®^ data, e.g., assessment of animal condition and avoidance distance, were recorded in the barn. 

Before we started with the on-farm assessment, a detailed methods training of the WQ^®^ protocol was conducted. The three different assessors experienced in dairy cattle before were trained by a qualified person with many years of experience in the assessment of the methodology of WQ^®^ protocol for dairy cattle. The intensive and detailed training was based on photographs and videos as well as exercises in dairy cow farms. As data were collected by different assessors, inter-observer reliability testing took place after each of the training courses which were held before the data-acquisition phases in winter and in summer. The results of the inter-observer reliability tests were in good to very good accordance for all animal-related indicators for all assessors.

### 2.2. Statistical Analysis

Statistical analyses were carried out with SAS^®^ 9.3 (procedure PROC MIXED, SAS Institute Inc., Cary, NC, USA). All parameters were evaluated at herd level by a mixed model with repeated measures. The fixed factors were group (Groups 0, 1 and 2), season (winter, summer) and their interaction (Group x season). A *p*-value of 0.05 was assumed as the significance limit. To verify the assumption of the models, residuals and homogeneity of variance were checked by the procedure PROC UNIVARIATE (SAS Institute Inc., Cary, NC, USA) and visually.

Three criteria of the WQ^®^ were excluded from the evaluation due to confounding effects (“expression of other behavior” because of circular reasoning, “thermal comfort” because no indicators has been developed, and “ease of movement” because of infinite likelihood, as the same data set for the two seasons was used). Consequentially, the aggregation into WQ^®^ principles or into a WQ^®^ overall score was not performed in our study because of the three missing WQ^®^ criteria.

## 3. Results

### 3.1. Measures

The results at the level of WQ^®^ measures are shown in Table 2. There was no effect on the body condition score; however, there were significant effects of grazing on two parameters of lying down behavior. The duration of lying down movements were lower in Groups 1 and 2 compared to Group 0, while there were hardly any differences between Groups 1 and 2. On average of all farms, lying down was quicker in summer than in winter. In winter, collisions during lying down occurred in the order of Group 1 < Group 2 < Group 0. Of course, no collisions were recorded for groups with access to pasture in summer.

We found an effect of season concerning the cleanliness of animals: Cows in summer were cleaner at the flank and upper legs compared to cows in winter. For the other body areas, no effects were found. Regarding integument alterations and locomotion disorders, grazing had an effect on the incidence of hairless patches and lameness. The percentage of cows with at least one hairless patch and no lesion was affected by grazing and season. There were fewer cows with hairless patches in summer than in winter, especially in Groups 1 and 2, but fewest in Group 2. There were less moderately and severely lame cows in Groups 1 and 2 than in 0. For both cases, Group 2 showed the lowest percentage of lame cows, followed by Group 1 and then Group 0.

Referring to diseases, only one group effect and three season effects were found. Namely, ocular discharge occurred in the order of Group 1 < Group 2 < Group 0. Furthermore, percentage of ocular discharge was higher in summer than in winter. The percentage of diarrhea was also higher in summer than in winter, whereas the percentage of vulvar discharge was lower in summer than in winter.

In the individual parameters (percentage of head butts and displacements) of social behavior, no effects of group or season were found.

### 3.2. WQ^®^ Criteria

In this section, results at WQ^®^ criteria level are only described if other effects, different from those outlined above (Section 3.1), occurred. 

For the criterion “absence of prolonged thirst,” we found an effect of season and an interaction between group and season (Table 3). The scores in summer were lower than in winter and, for Groups 1 and 2, in summer compared to winter. In Group 0, there was no difference between summer and winter for the former criterion. In the criterion “absence of pain,” farms with grazing had a better score compared to farms with minor/zero grazing, whereas Groups 1 and 2 had only marginal differences. Season had an effect as well: in summer, compared with winter, a better score was achieved. In contrast to the findings in Section 3.1, an interaction of Group x season was found for the criterion “expression of social behavior.” While in Groups 0 and 1 hardly any differences occurred between the seasons, a better score could be found in Group 2 for summer compared to winter. In the criterion “good human–animal relationship” farms in summer had an inferior score compared to winter. Moreover, farms with opportunity for grazing had a lower score in winter than in summer. This is in contrast to the farms with minor/zero grazing where scores were in the opposite direction. 

## 4. Discussion

The results of the present study are mainly in line with our first hypothesis: that grazing improves cows’ welfare. Additionally, we assumed that the effects would be clearer in regard to increasing daily grazing time, but this occurred only in certain measures.

Concerning the criterion “absence of prolonged hunger” consisting of the measure “% of very lean cows,” no positive effects of grazing were expected. Grazing may have negative effects on the nutritional status and may show as a poor body condition score or poor body weight [9,12,15,21]. Olmos et al. [14] found that cows in pasture-based systems had a higher nutritive metabolic stress and a lower rumen filling level compared to the cows with continuous housing with total mixed ration feeding. However, we found no differences between the groups or season in regard to the body condition score. Reasons for this could be the additional feeding of total mixed ration (TMR) for cows of all farms with grazing, except one farm, in our study. 

In the criterion “absence of prolonged thirst,” we found an unexpected effect of grazing. Farms with pasture during summer had a worse situation compared to winter and compared to farms with minor/zero grazing. These worse scores in Groups 1 and 2 were predominantly related to an insufficient supply of water (number of water troughs or bowls) on pasture, and caused by poor management, respectively. Only good management leads to high cow welfare [2]. In accordance, the European Food Safety Authority recommended “well managed pasture should be given for cows” [22].

The criterion “comfort around resting” was significantly and positively influenced by grazing. A seasonal effect was found, but there were no apparent differences between Groups 1 and 2. The duration of lying down movements and collision especially contributed to the better scores for Groups 1 and 2. These might be due to more comfortable lying conditions, e.g., softer lying surfaces on pasture. Similarly, Olmos et al. [5] concluded better comfort at pasture than in housing conditions. Previous investigations also showed improved lying behavior at pasture [6,23,24]. Furthermore, deprivation in lying comfort could have negative effects, e.g., more physically stress (higher cortisol level) in cows [25] following poorer welfare. Thus, an improved lying behavior results in good welfare.

In the criterion “absence of injuries,” farms with grazing achieved better scores than farms with minor/zero grazing. This good score was predominantly related to the low percentage of cows with hairless patches and of cows with lameness. These results are in line with previous investigations that grazing resulted in a reduction of locomotion disorders and improved integument condition (lameness [5,8,9,10]; integument [11,26]).

Regarding daily grazing time, farms with high grazing had a lower percentage of cows with hairless patches and fewer incidences of lameness compared to medium and minor/zero grazing farms. These findings are in line with Keil et al. [27] (only hock lesions) and Burow et al. [11] who found fewer hairless patches and fewer lesions located at the hock with higher grazing duration. While the reduced prevalence of hairless patches was influenced by season, the parameter lameness was independent in our study. The beneficial effect of pasture-based systems has indicated a positive long-term effect on lameness in other studies as well [2], with reduced incidence of lameness being recorded during the housed season of systems with pasture [8,28]. Our results were similar.

At the level of WQ^®^ criteria, we did not find effects of daily grazing time on the criterion “absence of disease,” but there was an effect on the level of WQ^®^ measures for “ocular discharge.” Ocular discharge occurred in herds with year-round indoor housing more often than in pasture-based systems, and the prevalence was higher in summer than in winter. These findings could be explained by increased incidence of flies, higher emissions of ammoniac, infiltration or dust in the barn. In total, this welfare problem did not occur very often, thus this indicator seems to be not very meaningful for our interpretation of results. The percentage of diarrhea was higher in summer than in winter. Burow et al. [11] found that feces consistency was thinner in summer than in winter. One reason for this could be an excess of protein and sucrose due to the fresh feeding of forage from pasture in summer. The percentage of vulvar discharge was lower in summer than in the winter housed period, possibly due to poorer hygienic conditions in the stable during the winter housed period, as also suggested by Sheldon et al. [29]. They described a risk of metritis by less hygienic conditions indoors and the associated contamination of uterine lumen.

Surprisingly, we found an effect of season on the criterion “absence of pain” in all groups, resulting in a better score in summer than in winter. These differences were predominantly caused by changes in the management of dehorning in Group 0. The reason for this increase was because of changes in the legal regulations of calf dehorning in several federal states of Germany in 2015, according to which the use of anesthetics and analgesics is generally required and also clearly defined. Groups 1 and 2 were less influenced by this fact, because the majority of these farms was under organic management, did not dehorn at all, or had already used appropriate measures.

The farms with high grazing stand out in the criterion “expression of social behavior,” because these farms had a better score in summer than in winter. One reason could be that the social behavior was recorded at pasture for this group, whereas for Group 1, the social behavior was assessed in the barn (behavior in both groups was assessed after afternoon milking). Nevertheless, cows which spent more time at pasture also had more space available over longer time periods. Expectedly, with more space allowance, less agonistic interaction (e.g., pushing, avoiding, and threatening) were found in cattle [30]. This is consistent with previous studies, which detected less agonistic behavior at pasture phases compared to the housing phases [6,31].

The criterion “good human–animal relationship” was affected by season. All groups in summer had a worse score than in winter, especially in farms with grazing. The authors of [32] detected a higher avoidance distance after the grazing period compared to the start of the grazing period and attributed the results to a changed management for cows during the summer with less contact with humans. 

Concerning “positive emotional state,” farms with grazing had a better score in summer than in winter, whereas for minor/zero grazing farms the contrary results were found. It is known from preference tests in dairy cattle that they spend more time at pasture when they are given free choice [33,34,35]. Here the cows had the opportunity to stay in the barn with TMR or at the pasture, and in all three studies the cows showed a clear preference for the pasture. In addition, access to pasture provides better opportunities for animals to act out their species-specific behavior compared to housing conditions [3]. Therefore, it is argued that the cows showed a better “positive emotional state” in the qualitative behavior assessment at pasture in our study. 

However, further studies with a higher number of involved farms are needed to examine the effects of grazing on animal welfare of dairy cows in organic and conventional farming. Furthermore, more research is required in the missing criteria of the WQ^®^ protocol for dairy cattle and to evaluate welfare of dairy cows at pasture.

## 5. Conclusions

The results at the WQ^®^ criteria level show an improvement for dairy cows in farms with grazing in five out of nine examined criteria. In line with our hypothesis, we found positive effects of grazing in general on the criteria “comfort around resting,” “absence of injuries,” “absence of disease,” “expression of social behaviors,” and “positive emotional state.” Concerning daily grazing time, we expected more pronounced effects of high grazing in these WQ^®^ criteria, but only several parameters, i.e., “% of cows with at least one hairless patch, not lesion”; “% of moderately lame cows” and “% of severely lame cows,” were affected positively at the level of WQ^®^ measures. Furthermore, the range of results in all groups varying from positive to negative WQ^®^ results occurred in all systems, regardless of the level of grazing. Therefore, the potential improvements of farms must always be detected at the level of the individual farm. Although grazing offers great potential to enhance welfare of dairy cows, beneficial effects are not guaranteed when management does not satisfy the animals´ needs. Our results show that the WQ^®^ protocol for dairy cattle can be used to detect the effects of daily grazing time on dairy cow welfare. A detailed definition in the WQ^®^ protocol of the circumstances under which measures should be assessed on pasture as opposed to the barn might serve comparability in future studies on this matter.

## Figures and Tables

**Table 1:** Overview of group allocation.

Group	Labeling	Daily Grazing Hours	Number of Farms		Total n	Further Information
			Organic	Conventional		
0	minor/zero grazing	0 to <6	3	11	14	thereof zero grazing n = 12, outdoor run n = 2 (one organic and one conventional)
1	medium grazing	≥6 to <12	7	3	10	
2	high grazing	≥12	5	3	8	thereof tethered n = 2 (both conventional)

**Table 2:** Animal-based WQ^®^ measures (mean (min-max)) for three groups (Groups 0, 1, and 2) and two seasons (W = winter and S = summer). Bold highlighted *p*-values are *p* < 0.05. Units are given in the description column.

WQ^®^ Measures	Group 0 (n = 14)		Group 1 (n = 10)		Group 2 (n = 8)		*p-Value*		
	W Season	S Season	W Season	S Season	W Season	S Season	*Group*	*Season*	*Group x Season*
% of very lean cows	7 (0–24)	6 (0–18)	6 (0–19)	7 (0–19)	6 (0–19)	9 (3–16)	*0.800*	*0.425*	*0.688*
D of lying down movements (s)	6 (4–9)	5 (4–6)	5 (4–7)	4 (3–5)	6 (4–9)	4 (3–4)	***0.010***	***<0.001***	*0.156*
% of lying cows which lie partly outside lying area	3 (0–30)	13 (0–90)	2 (0–11)	2 (0–11)	11 (0–44)	0 (0–0)	*0.357*	*0.922*	*0.070*
% of lying down movements with collisions	32 (0–71)	40 (0–80)	15 (0–50)	0 (0–0)	25 (0–67)	0 (0–0)	***<0.001***	*0.067*	***0.042***
% of cows with dirty lower legs	87 (54–100)	88 (58–100)	87 (60–100)	78 (13–100)	96 (87–100)	83 (24–97)	*0.484*	*0.140*	*0.401*
% of cows with dirty flank and upper legs	67 (9–100)	54 (11–96)	60 (35–100)	40 (14–80)	62 (30–100)	29 (9–71)	*0.122*	***0.001***	*0.435*
% of cows with dirty udder	38 (11–93)	35 (9–74)	31 (7–80)	32 (0–81)	36 (11–59)	11 (3–21)	*0.123*	*0.084*	*0.129*
% of cows with at least one hairless patch, no lesion	47 (35–70)	49 (30–76)	44 (13–65)	34 (3–53)	55 (21–70)	22 (3–33)	***0.038***	***0.001***	***0.001***
% of cows with at least one lesion	22 (7–40)	27 (4–63)	21 (3–38)	23 (2–45)	21 (12–35)	10 (0–21)	*0.094*	*0.691*	*0.128*
% of moderately lame cows	9 (2–22)	8 (0–18)	6 (0–13)	6 (0–31)	1 (0–5)	4 (0–9)	***0.004***	*0.857*	*0.622*
% of severely lame cows	2 (0–10)	2 (0–7)	1 (0–4)	1 (0–6)	0 (0–2)	0 (0–2)	***0.036***	*0.764*	*0.990*
% of cows with nasal discharge	16 (4–35)	13 (0–44)	6 (0–12)	11 (0–31)	9 (3–23)	7 (0–20)	*0.098*	*0.865*	*0.418*
% of cows with ocular discharge	3 (0–9)	7 (0–17)	1 (0–8)	3 (0–10)	1 (0–3)	3 (0–9)	***0.006***	***0.007***	*0.159*
FR of coughing per cow per 15 min	1 (0–2)	1 (0–2)	1 (0–2)	1 (0–2)	1 (0–1)	0 (0–0)	*0.271*	*0.075*	*0.188*
% of cows with increased respiratory rate	0 (0–0)	0 (0–2)	0 (0–0)	0 (0–0)	0 (0–3)	0 (0–0)	*0.468*	*0.516*	*0.189*
% of cows with diarrhoea	2 (0–18)	1 (0–6)	0 (0–2)	2 (0–9)	1 (0–9)	5 (0–14)	*0.186*	***0.041***	*0.155*
% of cows with mastitis	10 (0–19)	15 (4–30)	12 (0–30)	11 (0–28)	20 (7–40)	16 (3–34)	*0.081*	*0.883*	*0.202*
% of cows with vulvar discharge	1 (0–3)	0 (0–2)	1 (0–3)	0 (0–3)	1 (0–7)	0 (0–0)	*0.936*	***0.041***	*0.263*
% of dystocia	4 (0–11)	6 (0–20)	5 (0–17)	4 (0–24)	6 (0–14)	7 (0–20)	*0.630*	*0.809*	*0.653*
% downer cows	5 (1–8)	3 (0–8)	5 (1–11)	6 (0–17)	8 (0–19)	5 (0–17)	*0.081*	*0.883*	*0.202*
% of mortality	2 (0–6)	3 (0–7)	3 (0–10)	2 (0–5)	1 (0–3)	2 (0–3)	*0.229*	*0.860*	*0.561*
FR of butts per cow/hour	1 (0–2)	1 (0–2)	0 (0–1)	1 (0–4)	1 (0–2)	0 (0–1)	*0.474*	*0.873*	*0.156*
FR of displacements per cow/hour	0 (0–1)	0 (0–1)	0 (0–2)	0 (0–2)	0 (0–1)	0 (0–0)	*0.172*	*0.539*	*0.569*

% = percentage, D = duration, FR = frequency.

**Table 3:** Four WQ^®^ principles divided in twelve WQ^®^ criteria, scores (mean (min-max)) for three groups (Groups 0, 1, and 2) and two seasons (W = winter and S = summer). Highlighted in bold are *p*-values that are *p* < 0.05.

WQ^®^ Criteria	Group 0 (n = 14)		Group 1 (n = 10)		Group 2 (n = 8)		*p-Value*		
	W Season	S Season	W Season	S Season	W Season	S Season	*Group*	*Season*	*Group x Season*
**Good Feeding**									
1. Absence of prolonged hunger	67 (27–100)	68 (32–100)	70 (31–100)	64 (31–100)	71 (31–100)	53 (34–78)	*0.767*	*0.200*	*0.422*
2. Absence of prolonged thirst	47 (3–100)	48 (3–100)	62 (3–100)	12 (3–60)	62 (3–100)	30 (3–100)	*0.662*	***0.001***	***0.014***
**Good Housing**									
3. Comfort around resting	34 (0–56)	29 (0–54)	40 (9–54)	54 (35–71)	36 (16–54)	64 (54–78)	***0.007***	***<0.001***	***<0.001***
4. Thermal comfort	-	-	-	-	-	-	*-*	*-*	*-*
5. ease of movement	-	-	-	-	-	-	*-*	*-*	*-*
**Good Health**									
6. Absence of injuries	59 (29–81)	58 (30–73)	64 (45–90)	66 (44–95)	71 (62–84)	81 (71–91)	***0.003***	*0.126*	*0.097*
7. Absence of disease	41 (25–65)	39 (25–57)	42 (25–50)	49 (33–86)	38 (22–65)	42 (20–86)	*0.360*	*0.423*	*0.606*
8. Absence of pain	46 (20–100)	66 (28–100)	74 (28–100)	85 (52–100)	71 (49–100)	81 (49–100)	***0.034***	***0.001***	*0.436*
**Appropriate Behavior**									
9. Expression of social behaviors	70 (50–91)	72 (41–90)	72 (14–90)	68 (2–91)	69 (41–97)	88 (66–100)	*0.507*	*0.085*	***0.037***
10. Expression of other behavior	-	-	-	-	-	-	*-*	*-*	*-*
11. Good human–animal relationship	55 (37–86)	53 (28–74)	62 (33–88)	59 (34–89)	64 (32–84)	56 (30–90)	*0.535*	***0.038***	*0.469*
12. Positive emotional state	84 (69–95)	77 (44–96)	80 (58–97)	89 (70–97)	85 (77–96)	90 (85–94)	*0.110*	*0.357*	***0.016***

A value of 100 corresponds to the best and a value of 0 to the worst of all possible values, according to WQ^®^.
